# The Effect of Base Theory Educational Intervention on Health-Promoting Lifestyle in Women Susceptible to Cardiovascular Diseases: Application of the Theory of Planned Behavior

**DOI:** 10.1155/2023/8528123

**Published:** 2023-07-21

**Authors:** Peyman Izadpanah, Negin Saadat, Bahareh Kabiri, Fatemeh Mohammadkhah, Pooyan Afzali Harsini, Ali Khani Jeihooni

**Affiliations:** ^1^Department of Cardiology, School of Medicine, Namazi Teaching Hospital, Shiraz University of Medical Sciences, Shiraz, Iran; ^2^Department of Public Health, School of Health, Fasa University of Medical Sciences, Fasa, Iran; ^3^Department of Public Health, School of Health, Ilam University of Medical Sciences, Ilam, Iran; ^4^Department of Community Health, Child Nursing and Aging, Ramsar School of Nursing, Babol University of Medical Sciences, Babol, Iran; ^5^Department of Public Health, School of Health, Kermanshah University of Medical Sciences, Kermanshah, Iran; ^6^Nutrition Research Center, Department of Public Health, School of Health, Shiraz University of Medical Sciences, Shiraz, Iran

## Abstract

**Background:**

Cardiovascular diseases are the second leading cause of mortality, disability, and reduced productivity in women over 40 years and the first cause of mortality in women over 60 years. Therefore, the present study aimed to determine the effect of educational intervention based on theory of planned behavior (TPB) on health-promoting lifestyle in women susceptible to cardiovascular diseases.

**Methods:**

This quasiexperimental study was conducted on 200 women susceptible to cardiovascular diseases referred to health centers in Fasa city, Fars province, Iran. The available sampling was performed on women who referred to the centers and had a family record. In this study, two health-promoting lifestyle questionnaires consisting of 49 questions and the theory of planned behavior questionnaire consisting of 50 questions were used. The obtained data were analyzed by using the SPSS software version 24 in two stages before and six months after the educational intervention through paired *t*-test, independent *t*-test, chi-square test, and McNemar test.

**Results:**

The mean age of women in the experimental and control groups was 38.74 ± 9.22 and 39.14 ± 9.08 years, respectively. The results showed a significant increase in the experimental group after six months of intervention in terms of health-promoting lifestyle and constructs of the theory of planned behavior. Also, mean blood pressure, fasting blood sugar, and smoking of experimental group decreased six months after the educational intervention.

**Conclusion:**

Considering the irreplaceable role of education in adopting healthy behaviors and the role of women in strengthening the family foundation, quality educational programs should be designed and regularly implemented by health care providers for women.

## 1. Background

Cardiovascular diseases (CVDs) are the biggest threats to public health globally that will cause a major burden to the nation's economic, society, and health care system [1].

Following advances in the diagnosis and treatment of gynaecological and obstetric diseases, cardiovascular diseases are now the leading cause of death in women [2]. Women at younger ages have lower risk factors for cardiovascular diseases than men; however, in older ages, these risk factors are more common in men of the same age. In addition, the effect of diabetes, obesity, and increased fat on cardiovascular diseases in women is more significant [3].

According to the latest report of the World Health Organization in 2023, cardiovascular diseases (CVDs) are the leading cause of death globally, and 19.9 million people will die every year due to this disease which includes 23% of all deaths in the world and 85% were due to heart attack and stroke. Cardiovascular diseases (CVDs) are the leading cause of death globally. At least three-quarters of the world's deaths from CVDs occur in low- and middle-income countries. Also, out of the 17 million premature deaths (under the age of 70) due to noncommunicable diseases in 2019, 38% were caused by CVDs [4]. Iran, as a developing country with a low-income level, 26.4% of recorded deaths were due to cardiovascular diseases [5]. Iran has one of the highest age-standardized prevalence rates of cardiovascular diseases [6] and a high cardiovascular diseases mortality rate [7]. By 2025, the burden of the cardiovascular diseases in the country will be more than double the rate in 2005 [8]. The proper management of lifestyle-related modifiable risk factors reduces the rates of heart diseases [9]. Adherence to a healthy lifestyle may substantially lower the burden of cardiovascular diseases and reduce the risk of women [10]. Smoking is the leading cause of death for adults in developed countries, responsible for 22% of cardiovascular diseases in industrialized countries. It is also responsible for more than one in six deaths from cardiovascular diseases in the United States. It is estimated that smoking causes 630,000 deaths each year due to cardiovascular diseases in developed countries [11, 12]. Proper diet and physical activity are very important in order to prevent against CVD [13]. Obesity and overweight are other risk factors for cardiovascular diseases, so there is a significant relationship between increased BMI and increased triglycerides, cholesterol, blood sugar and blood pressure [14], and stroke [15]. Inactivity, which is the result of machine life and is one of the ten leading causes of death in the world, increases the risk of cardiovascular diseases by 2–5 times [16, 17]. The amount of regular physical activity recommended for adults is 30 minutes of moderate intensity throughout the week or at least 5 days a week, which plays a significant role in reducing the risk of chronic diseases including cardiovascular diseases [18]. Many existing studies address that the mentioned activities are all elements encompassed in health-promoting behaviors for CVD prevention. Health-promoting behaviors play a major role in many lifestyle-related diseases prevention. Therefore, understanding health-promoting behaviors improve their health and it is crucial for developing tailored health promotion interventions and thereby [19]. In the other hand, knowledge of behavioral risks is important in lifestyle change and individuals who perceive themselves at higher risk of cardiovascular disease are more likely to adopt a healthy lifestyle. Knowledge towards cardiovascular diseases risks and prevention is low in vulnerable communities [20]. Also, the perceived barriers, perceived benefits, family history of CVD, and screening intention enable people to engage in health-promoting behaviors related to cardiovascular disease prevention [19]. Health education professionals use different theories in healthcare professional education [21]. One of the most common theories in health education and health promotion is the theory of planned behavior, first proposed in the late 1980s by Martin Fishbin and Ajzan [22]. The TPB provides a useful framework for explaining and predicting health behaviors [23]. On the other hand, one of the theories that this theory is used to predict behavior and carry out educational intervention is the theory of planned behavior, which predicts a person's intention to perform a specific behavior [24, 25]. A prominent feature of this theory is the effect of behavioral intention on the behavior of individuals. This theory claims that behavioral intention precedes behavior and is determined by attitudes toward behavior and subjective norms. Attitudes toward behavior include a person's general desire or hatred for any particular behavior and are characterized by behavioral beliefs and outcome evaluation. Subjective norms are considered as a secondary predictor of behavioral intention, and a person who believes that certain people approve of behavior and are motivated to meet their expectations has positive subjective norms. Perceived behavioral control refers to an individuals' understanding of how much control they have over their voluntary actions [22]. The results of the studies show the effectiveness of this model in predict and promoting healthy behaviors related to lifestyle for the prevention of cardiovascular disease [26–28]. Therefore, this model has the ability to develop educational interventions to change unhealthy behaviors [29] and considering the importance of preventive behaviors in the prevention of cardiovascular disease. Due to the higher effectiveness of theory-based studies than routine studies in the prevention of cardiovascular diseases, this study aims to determine the effect of educational intervention based on the theory of planned behavior on health-promoting lifestyle in women susceptible to cardiovascular diseases in Fasa city, Fars province, Iran.

## 2. Methods

This quasiexperimental study was conducted on 200 women susceptible to cardiovascular diseases, who referred to health centers in Fasa city, Fars province, Iran. After obtaining the necessary permits from Fasa University of Medical Sciences and Fasa Health Center, by referring to selected centers (out of 6 health centers in Fasa, 2 centers were randomly selected) available sampling was performed among women who referred to these centers and had a family record. Then, the researcher introduced himself to participants and stated the purpose of the study, emphasizing the confidentiality of information.

According to a study by Babaei et al. [30], the sample size was considered 200. People were randomly divided into two control and control groups (100 in the experimental group and 100 in the control group). People at risk were those who met at least two of the five risk factors for cardiovascular diseases (smoking, blood pressure above 140.90 mm Hg, BMI above 24.9, known diabetes mellitus or FBS above 125 mg/dL, and total cholesterol 200 mg/dL/LDL above 130 mg/dL) [31]. Other criteria included minimum literacy, 18–65 years of age, no depression, and ability to perform daily activities.

Inclusion criteria were willingness to participate in the study, 20–50 years of age and no pregnancy. Exclusion criteria were the absence of more than two training sessions, unwillingness to participate in the study, and the occurrence of complications making it difficult for the individual to continue the study.

In this study, two questionnaires were used. The first one, Health Promoting Lifestyle Questionnaire consisted of 49 questions in 6 dimensions, including mental development (9 questions); health responsibility (8 questions); interpersonal relationship (8 questions); stress management (7 questions); physical activity (8 questions); and the nutrition (9 questions). The answers were based on a 4-point Likert scale with options always (3 points), often (2 points), sometimes (1 point), and never (0 points). The validity and reliability of the Persian version of this questionnaire was confirmed in a study by Issa Mohammadi Zeidi et al. in 2011. Cronbach's alpha was 0.82 for the tool. All cases had an acceptable case-total correlation (greater than 0.34). Test-retest results showed stability for the health-promoting lifestyle questionnaire and its subcategories. Confirmatory factor analysis of the model indicated an acceptable fit [32]. The second questionnaire, the TPB Questionnaire (questionnaire made by the researcher), which was prepared according to other studies [21, 26–28], consisted of 50 questions in 2 parts. The first part included demographic characteristics such as (age, weight, height, household size, education, marital status, occupation, monthly household income, diabetes, hypertension, family history of cardiovascular diseases, smoking and hookah use, and history of training related to cardiovascular diseases). The second part included the constructs of the TPB including attitude, subjective norms, perceived behavioral control, and behavioral intention.

Attitude was measured by 10 questions based on a Likert scale. The answers ranged from “strongly disagree” (scored 1) to “strongly agree” (scored 5). The perceived behavioral control was measured by 8 questions based on a Likert scale. The answers ranged from “strongly disagree” (scored 1) to “strongly agree” (scored 4). Subjective norms were measured by 6 questions based on a Likert scale. The answers ranged from “never” (scored 1) to “always” (scored 3). Behavioral intention was measured by 6 questions based on a Likert scale. The answers ranged from “strongly disagree” (scored 1) to “strongly agree” (scored 3).

The validity of the items was evaluated by calculating the item impact score index, higher than 0.15, the content validity ratio index, higher than 0.79. To determine the face validity of the tool, a list of compiled items was targeted by 30 women with demographic, economic, and social characteristics similar to the population. To determine the validity of the content, the opinions of 10 experts (outside the research team) on health education and health promotion, 1 nutritionist, and 1 cardiologist were used. Using the Lawshe index, items higher than 0.56 were considered essential and kept for further analysis. Most of the items were above 0.70. Based on Cronbach's alpha, the overall reliability was calculated to be 0.89. Also, reliability of attitude, subjective norms, perceived behavioral control, and behavioral intention was calculated to be 0.84, 0.89, 0.82, and 0.87, respectively.

The height of the participants was measured using a tape measure fixed on the wall, in a standing position without shoes. Weight was measured with minimum dress and without shoes using a digital scale. Finally, body mass index (BMI) was calculated using SPSS software. It was considered as lean, normal, overweight, and obese equal to <18.5, 18.5–24.9, 25–29.29, and ≥30, respectively.

Systolic and diastolic blood pressure were measured before and 6 months after the educational intervention. All measurements in both experimental and control groups were performed by one of the researchers using the same blood pressure monitor.

Blood glucose levels were measured before and 6 months after the educational intervention. Physical activity was also measured self-reportedly.

Educational intervention for the experimental group based on the pretest results included 10 training sessions of 50–55 minutes through lectures, group discussions, questions and answers, videoclips, images, and PowerPoint. The training program was implemented by a PHD of health education and health promotion, a cardiologist, and a nutritionist. The content of the training sessions was concepts related to controlling blood pressure, blood sugar, blood lipids, and behaviors promoting health and preventing cardiovascular diseases. Also, in each training session, participants were assessed clinically (blood pressure, heart rate, blood oxygen, blood sugar, and weight). Also, an educational booklet was provided to the participants. Also, participants were provided videos on how to measure blood sugar, blood pressure, and educational information on obesity, diabetes, cardiovascular risk factors, and a healthy lifestyle and physical activity. One of the training sessions was held with the presence of a family member (preferably spouse), a doctor and staff of health centers as effective subjective norms.

Training sessions were held for ten groups of ten (100 people in the experimental group), one session per week. A WhatsApp group was formed to exchange information and an educational and motivational message was sent to patients every 5 days. Two follow-up sessions were held two months and four months after the intervention. The details of the training sessions are presented in Table 1.

Both experimental and control groups participated from the beginning to the end of the study and no one was excluded from the study. The control group did not receive any training program and were only invited to a special session to complete the questionnaire. At the end of the study, to comply with ethical standards, a training session was held for the control group. The questionnaire was completed by the experimental and control groups before and six months after the educational intervention.

## 3. Data Analysis

Data were entered using the SPSS24 software. Related descriptive and analytical tests have been used to analyze the data (*p*=0.05). To check whether the two groups were similar in terms of demographic variables, chi-square test and McNemar tests were used (*p*=0.05). Also, for the comparison of the mean score of constructs of the theory of planned behavior and dimensions of health-promoting lifestyle questionnaire in two groups before and six months after the educational intervention used of paired *t*-test (*p*=0.05) and for the comparison of the mean score of constructs of the theory of planned behavior and dimensions of health-promoting lifestyle questionnaire between two groups before and six months after the educational intervention used of independent *t*-test (*p*=0.05).

## 4. Results

In this study, 200 women susceptible to cardiovascular diseases participated. The mean age of women in the experimental and control groups was 50.74 ± 9.22 and 51.14 ± 9.08 years (*p*=0.206), respectively. The mean household dimension in the experimental and control groups was 3.79 ± 2.72 and 3.82 ± 2.67 years (*p*=0.214), respectively.

Based on the chi-square test, there was no significant difference between the experimental and control groups in terms of occupation, household income, education, marital status, diabetes, hypertension, family history of cardiovascular diseases, and history of training related to cardiovascular diseases (Table 2).

The results showed that before the educational intervention, there was a significant difference between the experimental and control groups in terms of attitude, subjective norms, and behavioral control; however, 6 months after the intervention, the experimental group showed a significant increase in each of the mentioned constructs (Table 3).

The results showed that before the educational intervention, there was no significant difference between the experimental and control groups in terms of dimensions of health-promoting lifestyle questionnaire; however, six month after the intervention, the experimental group showed a significant increase in the mentioned dimensions (Table 4).

No significant difference was observed in the mean blood pressure of the experimental and control groups before the intervention; however, the mean blood pressure (both systolic and diastolic) in the experimental group significantly decreased after the educational intervention. The results also showed that the blood sugar level in the experimental group showed a significant decrease after the educational intervention. Also, the average consumption of tobacco in the experimental group significantly decreased after the intervention (Table 5).

Comparison of the mean body mass index in the experimental and control groups showed that after the intervention, no significant difference was observed between the two groups.

## 5. Discussion

The aim of this study was to determine the effect of educational intervention based on theory of planned behavior (TPB) on health-promoting lifestyle in women susceptible to cardiovascular diseases.

Results of a recent study showed that after the educational intervention, the mean score of attitude toward healthy behaviors among women susceptible to cardiovascular diseases in the experimental group was significantly increased compared to the control group which is in a good agreement with the results of Zhao et al. [33], Shakibazadeh et al. [34], and Joveini et al. [35]. Discussing in groups and presenting positive and negative experiences caused participants to be more interested in patterning for taking healthy behaviors in preventing cardiovascular diseases. Zhao et al.'s study with the aim of investigating the effect of TPB-based intervention on the smoking behavior of Chinese high school students showed an increase in subjects' attitudes after the educational intervention [33]. Also, Shakibazadeh et al.'s study with the aim of investigating the effectiveness of an educational intervention using theory of planned behavior on health care empowerment among married reproductive-age women showed an increase in subjects' attitudes after the educational intervention [34].

Joveini et al.'s study with the aim of investigating the effects of an educational intervention on male students' intention to quit water pipe smoking: an application of the theory of planned behavior (TPB) and health action process approach (HAPA) showed an increase in subjects' attitudes after the educational intervention [35].

In this study, after the educational intervention, the mean score of Perceived behavioral control for healthy behaviors among women susceptible to cardiovascular diseases in the experimental group was significantly increased compared to the control group which is in a good agreement with the results of Zhao et al. [33], Alami et al. [36], and Horne et al. [37].

Zhao et al.'s study with the aim of investigating the effect of TPB-based intervention on the smoking behavior of Chinese high school students showed an increase in subjects' perceived behavioral control after the educational intervention [33].

Alami et al.'s study with the aim of investigating the effect of educational intervention on iron and vitamin D consumption based on the theory of planned behavior in Iranian adolescent girls showed an increase in subjects' perceived behavioral control after the educational intervention [36] and in Horne et al. The study with an aim of exploring attitudes, subjective norms, and perceived behavioral control in a genetic-based and a population-based weight management intervention showed an increase in subjects' perceived behavioral control after the educational intervention [37].

In this study, in each training session, participants were assessed clinically (blood pressure, heart rate, blood oxygen, blood sugar, and weight). Participants were provided videos on how to measure blood sugar, blood pressure, and educational information on obesity, diabetes, cardiovascular risk factors, and a healthy lifestyle and physical activity which can increase the structure of perceived behavioral control after the educational intervention.

In this study, after the educational intervention, the mean score of subjective norms for healthy behaviors among women susceptible to cardiovascular diseases in the experimental group was significantly increased compared to the control group. In this study, the training sessions was held with the presence of a family member (preferably spouse), a doctor and staff of health centers as effective subjective norms which is in a good agreement with the results of Ohnmacht et al. [38], Khani Jeihooni et al. [39], Moridi et al. [40], and Lareyre et al. [41].

Ohnmacht et al.'s study with the aim of investigating the pointers to interventions for promoting COVID-19 protective measures in tourism showed an increase in subjects' subjective norms after the educational intervention [38]. Also, Khani Jeihooni et al.'s study, with the aim of investigating the effect of educational intervention based on the theory of planned behavior (TPB) on doing breast self-examination in a sample of Iranian women, showed an increase in subjects' subjective norms after the educational intervention [39] and in Lareyre et al. study with the aim of investigating the characteristics and impact of theory of planned behavior interventions on smoking behavior showed an increase in subjects' subjective norms after the educational intervention [40].

The results of a recent study showed that after the educational intervention, the mean score of behavioral intention of the experimental group increased significantly compared to the control group, indicating the effectiveness of the used model. Our educational solution to improve the behavioral intention in preventing cardiovascular diseases was forming a WhatsApp group and providing educational-motivational messages. This was consistent with the results of studies by Chen et al. [42], Singh et al. [43], and Lin and Roberts [44]. In Chen et al.'s study with the aim of investigating the effects of perceived benefit on Vitamin D Supplementation Intention showed an increase in subjects' behavioral intention after the educational intervention [42]. Also, in Singh et al.'s study with the title of educational intervention of intention change for consumption of junk food among school adolescents in Birgunj metropolitan city, Nepal, based on theory of planned behaviors showed an increase in subjects' behavioral intention after the educational intervention [43], and in the study by Lin and Roberts with the title of using the theory of planned behavior to predict food safety behavioral intention showed an increase in subjects' behavioral intention after the educational intervention [44]. In this study, a WhatsApp group was formed to exchange information and an educational and motivational message was sent to patients every 5 days. Two follow-up sessions were held two months and four months after the intervention that can increased in subjects' behavioral intention after the educational intervention. Based on the theory of planned behavior, the best way to predict people's behavior is to observe their intentions. Intention means planning for a specific behavior [39]. Behavioral intentions will have the greatest impact on behavior change when the situation pattern is common and the behavior is observed specifically in this situation. However, for many health-promoting behaviors, the behavior is seen in a variety of situations and is not always common [27, 45].

Lifestyle is one of the most important determinants of health. According to studies, 60% of quality of life is related to lifestyle [46]. It is also reported that 53% of causes of death are related to the lifestyle. Many health problems such as obesity, cardiovascular diseases, various cancers, and addictions are associated with lifestyle changes [46–48].

The results of the present study showed that before the educational intervention, there was no significant difference between the experimental and control groups in terms of dimensions of health-promoting lifestyle (mental development, health responsibility, interpersonal relationships, stress management, physical activity, and nutrition); however, 6 months after the intervention, the experimental group showed a significant increase in each of the mentioned dimensions. In the study by Arrizabalaga-López et al. [49], nutrition and physical activity had the least and highest mean score, respectively.

Also, in a study by Wei et al., interpersonal relationships and health responsibility had the least and highest mean score, respectively [50].

Important points in this study were significant changes in stress management following training the relevant methods and techniques, including problem-solving skill and focusing on positive thoughts. In Xie et al.'s study, there was a significant correlation between physical activities and stress management and relief [51].

Also, Lopez and Klainin-Yobas, mention applied the theoretical tenets of the TPB to stress reduction [52, 53]. Also, in this study, educational methods such as group discussion and using educational clips on proper nutrition could create the highest positive changes in nutrition. In a study by Wang et al.'s, the seniors had better nutrition and eating habits than freshmen, which could be attributed to an increase in knowledge and awareness [54].

In this study, during the two stages before and six months after the educational intervention and using self-report tools, the amount of physical activity was evaluated and the results showed that before the intervention, the two groups did not have a statistically significant difference in daily physical activity; however, after educational intervention, a statistically significant difference was observed between the two groups. This is consistent with the results of studies by Al-Ali and Haddad [55]; these study educational methods such as group discussion and using educational clips on physical activity could create the highest positive changes in physical activity. Another goal of the educational intervention in this study was to reduce the mean level of fasting blood sugar, blood pressure, and BMI over a period of six months. The results showed a decrease in mean fasting blood sugar and improved blood pressure in women; however, no significant change was observed in the experimental group in terms of BMI. This is consistent with the results of studies by Kuller et al. [56] and Davis et al. [57], and in this study, educational methods could create the highest positive changes in level of fasting blood sugar and blood pressure. In the present study, smoking was not very common among women, similar to the results of studies in Mediterranean countries [58]. After educational intervention, smoking was reduced in the experimental group, indicating the effectiveness of the used model.

## 6. Conclusion

TPB-based intervention in changing individual behavior led to the improvement of lifestyle dimensions, TPB constructs, and clinical outcomes. In order to have a broad impact on creating sustainable change, it is suggested to use theories of behavioral change that also consider interpersonal, organizational, and social factors ecologically. One of the strengths of the current study is the theory-based research and need-based intervention in women susceptible to cardiovascular diseases. The present study was conducted on women susceptible to cardiovascular diseases in Fasa, the results will not be generalizable to other women in different ethnicities of the country; this could be one of the limitations of the study. Also, another limitation of the present study is the use of self-report tools in measuring the effectiveness of educational interventions (especially in daily physical activity and smoking). Considering the important role of women in strengthening family foundation, it is suggested that quality educational programs be always planned and implemented through health care providers. Also, it is suggested to healthcare authorities take steps to create and promote health-promoting lifestyle in women susceptible to cardiovascular diseases by considering relevant theory-based training programs, using related educational materials for women, as well as executive guidelines and protocols of healthcare centers. First, a healthy lifestyle should be defined for the members of the society so that all the members of the society reach a single point of view on the subject, and then research and educational programs related to the lifestyle should be expanded and appropriate policies with that lifestyle should be approved. In promoting a healthy lifestyle, there should be media coordination, and initiatives that have a health-oriented perspective for society should be supported, and all internal and external facilities should be used to expand and modify the lifestyle and create social structures, especially nongovernmental organizations, to support lifestyle modification should be mobilized.

## Figures and Tables

**Table 1:** Description of educational sessions in the experimental group.

Session number	Content	Educational methods	Time	Construct of TPB model
1–3	The content of the training sessions was concepts related to controlling blood pressure, blood sugar, blood lipids, and behaviors promoting health and preventing cardiovascular diseases. The training sessions were held once a week in groups of 10 people, and the people in these groups learned the material through group discussion and question and answer, thereby trying to change their attitude	Lectures, group discussions, questions and answers, videoclips, images, and PowerPoint, educational booklet and videos, WhatsApp group	50–55 minutes	Knowledge and attitude
4	The training sessions was held with the presence of a family member (preferably spouse), a doctor and staff of health centers as effective subjective norms		50 minute	Subjective norms
5-6	The content of the training sessions was concepts related to controlling blood pressure, blood sugar, blood lipids, and behaviors promoting health and preventing cardiovascular diseases. Also, in each training session, participants were assessed clinically (blood pressure, heart rate, blood oxygen, blood sugar and weight). Participants were provided videos on how to measure blood sugar, blood pressure, and educational information on obesity, diabetes, cardiovascular risk factors, and a healthy lifestyle and physical activity		50–55 minutes	Perceived behavioral control
7–10	A WhatsApp group was formed to exchange information and an educational and motivational message was sent to patients every 5 days. Two follow-up sessions were held two months and four months after the intervention		50–55 minutes	Behavioral intention and behavior

**Table 2:** Comparison of demographic characteristics of women participated in the study.

Variables		*Experimental group*		*Control group*		*p* value
		Number	Percentage	Number	Percentage	
Marital status	Single	8	8	10	10	*p*=0.197
	Married	78	78	80	80	
	Divorced	8	8	6	6	
	Widowed	6	6	4	4	

Education	Illiterate	0	0	0	0	*p*=0.201
	Primary school	11	11	8	8	
	Secondary school	25	25	27	27	
	High school	42	42	45	45	
	College	22	22	20	20	

Monthly income	Less than 30 million rials	45	45	40	40	*p*=0.180
	30–60 million rials	37	37	38	38	
	More than 60 million rials	18	18	22	22	

Occupation	Housewife	76	76	70	70	*p*=0.207
	Employed	24	24	30	30	

Diabetes	Yes	33	33	28	28	*p*=0.177
	No	67	67	72	72	

Hypertension	Yes	35	35	30	30	*p*=0.159
	No	65	65	70	70	

Family history of cardiovascular diseases	Yes	36	36	68	68	*p*=0.186
	No	64	64	12	12	

History of trainings related to cardiovascular diseases	Yes	16	16	88	88	*p*=0.258
	No	84	84	68	68	

**Table 3:** Comparison of mean score of TPB model structures in the experimental and control group before and after intervention.

Variables	Group	Before the intervention	The intervention 6 months after	Mean difference	*p* value
Attitude	Experimental	22.28 ± 4.30	40.68 ± 4.03	18.4	0.001
	Control	24.10 ± 4.14	27.55 ± 4.20	3.45 ± 0.06	0.117
	*p* value	0.198	0.001		

Subjective norms	Experimental	13.84 ± 2.76	24.52 ± 2.43	10.68 ± 0.33	0.001
	Control	14.28 ± 2.18	15.55 ± 2.41	1.27 ± 0.23	0.225
	*p* value	0.217	0.001		

Perceived behavioral control	Experimental	19.14 ± 3.30	34.07 ± 3.28	14.93 ± 0.02	0.001
	Control	18.88 ± 2.43	19.71 ± 2.45	0.83 ± 0.02	0.214
	*p*value	0.222	0.001		

Behavioral intention	Experimental	13.56 ± 2.14	26.74 ± 2.84	13.18 ± 0.7	0.001
	Control	13.97 ± 2.26	15.06 ± 2.27	1.09 ± 1.01	0.109
	*p* value	0.277	0.001		

**Table 4:** Comparison of the mean score of dimensions of health-promoting lifestyle in the experimental and control groups before and after intervention.

Variables	Group	Before the intervention	6 months after the intervention	Mean difference	*p* value
Mental development	Experimental	16.51 ± 2.09	12.26 ± 2.01	4.25 ± 0.08	*p* < 0.001
	Control	14.88 ± 2.44	15.04 ± 2.42	0.16 ± 0.02	*p*=0.354
	*p* value	0.102	0.001		

Health responsibility	Experimental	13.52 ± 1.86	19.82 ± 1.37	6.3 ± 0.49	*p* < 0.001
	Control	14.22 ± 1.69	15.01 ± 1.60	0.76 ± 0.09	*p*=0.235
	*p* value	0.128	0.001		

Interpersonal relationships	Experimental	14.32 ± 1.50	19.38 ± 1.57	5.06 ± 0.07	*p* < 0.001
	Control	14.85 ± 1.56	15.08 ± 1.92	0.23 ± 0.36	*p*=0.322
	*p* value	0.369	0.001		

Stress management	Experimental	9.74 ± 1.44	17.25 ± 1.35	7.51 ± 0.09	*p* < 0.001
	Control	10.02 ± 1.38	10.51 ± 1.34	0.49 ± 0.04	*p*=0.380
	*p* value	0.342	0.001		

Physical activity	Experimental	12.94 ± 1.53	19.48 ± 1.60	6.54 ± 0.07	*p* < 0.001
	Control	13.70 ± 1.47	14.12 ± 1.40	0.42 ± 0.07	*p*=0.312
	*p* value	0.136	0.001		

Nutrition	Experimental	14.38 ± 2.13	23.14 ± 2.10	8.78 ± 0.03	*p* < 0.001
	Control	14.05 ± 2.38	14.62 ± 2.36	0.57 ± 0.02	*p*=0.309
	*p* value	0.406	0.001		

**Table 5:** Comparison of mean blood pressure, blood sugar, and smoking in the experimental and control groups before and after intervention.

Variables	Group	Before the intervention	The intervention 6 months after	Mean difference	*p* value
Systolic blood pressure	Experimental	131.41 ± 15.67	121.02 ± 14.58	10.39 ± 1.09	*p* < 0.001
	Control	132.16 ± 15.82	132.60 ± 15.68	0.44 ± 0.14	*p*=0.160
	*p* value	0.166	0.001		

Diastolic blood pressure	Experimental	80.72 ± 9.37	74.36 ± 7.65	80.72 ± 1.72	*p* < 0.001
	Control	82.36 ± 9.24	82.57 ± 9.29	0.21 ± 0.05	*p*=0.361
	*p* value	0.082	0.001		

Blood sugar	Experimental	138.8 ± 65.4	127.6 ± 60.7	11.2 ± 4.7	*p* < 0.001
	Control	140.5 ± 68.6	138.4 ± 64.5	2.1 ± 4.1	*p*=0.348
	*p* value	0.338	0.001		

Tobacco use	Experimental	7.12 ± 3.75	6.08 ± 3.69	1.04 ± 0.06	*p* < 0.001
	Control	6.83 ± 3.90	6.86 ± 3.91	0.03 ± 0.01	*p*=0.386
	*p* value	0.184	0.001		

## Data Availability

The data used to support the study are available from the corresponding author upon request.

## Abbreviations

TPB: Theory of planned behavior.
